# Antibiotic prophylaxis in foot and ankle surgery: a systematic review of the literature

**DOI:** 10.1186/s13047-018-0303-0

**Published:** 2018-11-15

**Authors:** Mr Ravi Krishān Modha, Chris Morriss-Roberts, Madeleine Smither, Jonathan Larholt, Ian Reilly

**Affiliations:** Department of Podiatric Surgery, Ilkeston Hospital, Heanor Road, Ilkeston, Heanor, DE7 8LN UK

**Keywords:** Antibiotic prophylaxis, Foot surgery, Foot and ankle surgery, Antibiotic chemoprophylaxis, Surgical site infection, Postoperative infection, Podiatric surgery, Orthopedic surgery, Biofilm

## Abstract

**Background:**

With the advent of bacterial resistance, it is important now more than ever to evaluate use of antibiotic chemoprophylaxis in foot and ankle surgery. Within this area of the body there may be less dissection, surgery time with smaller incisions and importantly smaller sizes of implanted fixation as compared to other bone and joint procedures. Our objective was to systematically evaluate the quality of evidence behind existing guidelines.

**Methodology:**

A systematic literature search was performed: MEDLINE, CINHAL, EMBASE and the Cochrane library from 1990 up to March 2018. To avoid omitting any studies on the subject, Google Scholar was also used. The inclusion criterion were studies exploring perioperative antibiotic use, postoperative infection rates in elective foot and ankle surgery and studies associated with this subject evaluating antibiotic use in clean elective foot and ankle surgery. The exclusion criterion being studies upon contaminated or dirty surgery or those which were inclusive of procedures proximal to the foot and ankle.

**Results:**

Overall 11 studies met the inclusion criteria. From the grading of evidence, 2 level one and 4 level two studies were recognised. These studies ranked relatively highly in comparison to 5 studies that were graded as level three and level four tiers of evidence. Results of SSI rates found within this systematic review ranged from 0% to 9.4% of overall postoperative infections encountered after foot and ankle surgery in the studies analysed.

**Conclusion:**

Whilst fragmented, aspects of antibiotic chemoprophylaxis are established fields in elective surgery with a growing body of evidence. Evidence for antibiotic use however, specifically within elective foot and ankle surgery, is lacking. This systematic review is a seminal paper which delivers an impression of the most influential literature within the field of foot and ankle surgery, with the aim being to entice conclusions and guide future research.

## Background

Antimicrobial stewardship is central to attaining optimal clinical outcomes in surgery, diminishing adverse drug events, reducing cost burdens associated with Surgical Site Infection (SSI) and curtailing the pathogenesis of antimicrobial resistant strains. The vast expenditure and misapplication of antibiotic agents acts to hasten the advent of antibiotic resistant pathogens, rapidly becoming a global public health issue [1].

Patients infected with antibiotic resistant organisms are more likely to have lengthier, more costly inpatient stays and a greater frequency of morbidity and mortality [2–4]. It is documented that patients who do not receive appropriate antibiotic prophylaxis are 2.32 times more likely to acquire a SSI as compared to those who receive antibiotic chemoprophylaxis [5].

In orthopedic surgery, routine antibiotic prophylaxis is considered the standard of care, particularly within total hip and knee arthroplasty, trauma procedures and open fracture repair [6]. A Cochrane Review of SSI rate and antibiotic prophylaxis in bone & joint surgery, confirmed the effectiveness of prophylaxis in the prevention of both superficial and deep postoperative infection [7]. Data from 8447 participants in 23 studies evaluating closed fracture fixation, found a bolus dose of chemoprophylaxis significantly reduced SSI rate (risk ratio 0.40, 95% CI 0.24 to 0.67).

Comparatively, a recent review article examined the current evidence for antibiotic prophylaxis in hand surgery (where incision and fixation size may be comparable to the foot) with the recommendations that antibiotic prophylaxis should not be routinely used with patients without autoimmune disease, not taking steroid medication, in clean and hygienic patients or in procedures without hardware, of low haematoma risk and performed in an outpatient or ambulatory setting [8]. It has been suggested that in clean elective hand surgery, sterile prepping and draping technique is more pertinent to reducing the risk of postoperative infection and has fewer adverse effects than administration of antibiotic prophylaxis [9].

Nonetheless, antibiotic prophylaxis is adopted in clean elective hand surgery. An international survey revealed 49% of American surgeons and 13% of international surgeons gave antibiotics perioperatively for carpal tunnel release surgery, a procedure which does not require the use of fixation [10].

Within clean elective foot and ankle surgery the accepted rate of SSI quoted in the literature ranges between 0.26 to 2% [11] and the latest National Institute for Health and Clinical Excellence (NICE) guidelines for SSI state at least 5% of patients will suffer a postoperative infection following surgery [2]. The many heterogeneous studies with varying research methodologies, patient selection, procedure technique and local policy may account for the difference in SSI rate, though the importance of antibiotic choice and dosing may also play a significant role when equating these surgical outcomes [12].

The NICE Clinical Guideline 74 [2] states that for bone and joint surgery involving implantation of foreign material such as joint replacement prostheses and metalwork fixation, pharmacological choice for chemoprophylaxis should follow local policy, which are developed via national guidelines. This should be in line with patient ASA grade (American Society of Anaesthesiologists); current best practice; local resistance patterns; and type of surgery undertaken.

Comparably, the Society for Healthcare Epidemiology of America (SHEA), the Infectious Diseases Society of America (IDSA), the Surgical Infection Society (SIS), the American Society of Health-System Pharmacists (ASHP) advise several important factors to consider in SSI risk [3]:

- The health status of the patient, a ASA score of more than 2 denotes increased risk.
- Implantation of prosthetic fixation or device and case duration.
- Class of surgery; dirty contaminated or clean.

The NICE Clinical Guideline 74 [2] is based on a 1–10 panel rating system, conferred by medical professionals. In the process of development, proposals receiving a score of over 7.5, were published as central recommendations within the NICE guidelines. Eleven health professionals comprised the team of raters, however a surgeon undertaking any type bone and joint surgery was not represented on the panel of raters.

Nonetheless, the 2008 SIGN 104 Guidelines [4] and the 2013 ASHP report [3] advocate very similar practice as the NICE Clinical Guideline 74 [2] and have both critically appraised an abundance of literature through peer review in the formation of their respective recommendations. Methodical analyses of the available literature were utilised in order to apply findings to chemoprophylactic antibiotic use, in a wide variety of surgical specialties [3, 4].

Within foot and ankle surgery in the United Kingdom the PASCOM-10 database is used within the Podiatric Medicine profession to audit clinical performance, practice and outcomes [11]. Statistics reported from the database across 75 centres from 2010 to 2014 yielded an infection rate of 2.34% (suspected) and 0.67% (proven) in 11,673 patients undergoing scarf osteotomies with internal fixation. Interestingly, only 37.4% of patients were prophylaxed with antibiotics [13]. Based on the national database records therefore, it can be deduced that it is common practice in UK Podiatric Surgery to not use antibacterial prophylaxis in clean elective foot and ankle surgery where internal fixation is utilised, and/or where uninfected bone is involved [13]. These statistics remain consistent with what the national guidelines and literature advise as acceptable SSI rates and yet over 60% of patients in this cohort did not receive antibiotic prophylaxis.

The preponderance of literature concerning bone and joint surgery recounts procedures more proximal to the ankle and often with more substantial hardware, larger incisions and longer exposure times [2–4]. The SIGN and NICE guidelines particularly have not referenced any studies pertaining to surgery of the foot and ankle in their recommendations [2, 3]. Therefore, these recommendations are based on heterogeneous data that is extrapolated from other surgical procedures unrelated to the foot and ankle and as such the perceived strength of these recommendations is low.

It can be concluded that there is a necessity for new high level data related to antibiotic prophylaxis in foot and ankle surgery. A pre-requisite being analysis of the existing data, of which an exhaustive systematic review does not exist to date. Thus serving to increase not only the amount but also quality of available evidence on which to make practice recommendations with greater confidence [2–4].

## Methodology

To undertake this review a systematic literature search utilising the following electronic bibliographic databases was performed: MEDLINE (Medical Literature Analysis and Retrieval Online, Bethesda, MD), CINHAL (Cumulative index to Nursing and Allied Health Literature, Ipswich, MA) and EMBASE (ExerptaMedica Database, Amsterdam, Netherlands) the Cochrane library without date restriction up to 1st March 2018. To avoid omitting any studies on the subject, Google Scholar search engine was also used, as well as a hand search of all references listed at the end of each of the selected studies for review.

The keywords and medical subject heading (Mesh) terms used are shown in Table 1 with the Boolean operators being AND or OR.

**Table 1:**
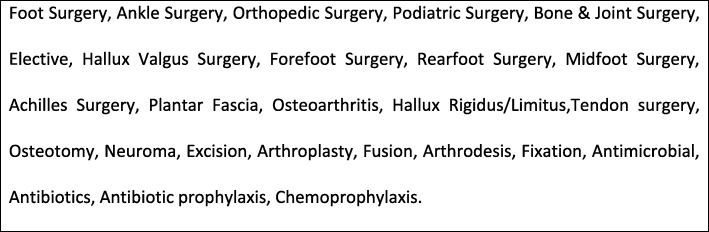
Search terms for formal literature search

The following (Table 2) represents the selection inclusion and exclusion criteria for the purposes of this systematic review, with careful thought to what variables would help to satiate the scarcities in the current guidelines outlined and how best those disputes raised could be tackled.

**Table 2:** Selection criteria for review

Inclusion Criteria for Studies:	
• International studies exploring perioperative antibiotic use and subsequent postoperative infection rates and complications in elective foot and ankle surgery • Studies analysing the effect of IV, IM and oral antibiotic prophylaxis in foot and ankle surgery • Studies analysing the effect of tourniquet application in conjunction with antibiotic prophylaxis in foot and ankle surgery • Studies published in English • Studies published between 1990 and 2018	
Exclusion Criteria for Studies:	
• Studies which included more proximal lower limb procedures in their methodology or findings e.g. knee or hip surgery • Studies outside the date range • Studies which included specific patient cohorts only e.g. Type 1 diabetics only	

The preliminary search yielded a total of 135 journal articles conducted by the primary author (RKM) and repeated by a secondary author (MS). Publication dates ranged from 1990 to 2018. The two screeners (RKM & MS) then reviewed the titles, abstracts and full texts of relevant articles for their eligibility based on the defined inclusion and exclusion criteria. Any disputes or cases of ambiguity were settled by a third screener (JL). This process led to the exclusion of 96 studies which clearly did not meet the inclusion criteria of the review. From the remaining 41 studies, 30 were excluded after examination and assessment of eligibility, as they too did not meet the inclusion criteria as first thought. The primary reasons being, that the majority included hip or knee surgery within their reviews and were not exclusive to surgery of the foot and ankle. Nine studies were deemed to meet the eligibility criteria. The references of inclusive studies were screened and a further 2 full text articles were identified. Eleven studies were included in the final review, which were then reviewed using the PICO tool [14, 15] to extract the relevant information needed to answer the research question in a systematic fashion thus reducing error and bias [16]. All studies were then collated and classified, with recommendations then graded A to C based on the level of associated evidence formulated by two internationally accepted hierarchies of evidence (see Tables 3, 4) [2].

**Table 3:** Classification of evidence

Level	Source of Evidence
1++	High-quality meta-analyses, systematic reviews of RCTs, or RCTs with very low risk of bias
1+	Well-conducted meta-analyses, systematic reviews of RCTs, or RCTs with a low risk of bias
1−	Meta-analyses, systematic reviews of RCTs, or RCTs with a high risk of bias
2++	High-quality systematic reviews of case-control or cohort studies; high-quality case-control or cohort studies with a very low risk of confounding, bias, or chance and a high probability that the relationship is causal
2+	Well-conducted case-control or cohort studies with a low risk of confounding, bias, or chance and a moderate probability that the relationship is causal
2−	Case-control or cohort studies with a high risk of confounding, bias, or chance and a significant risk that the relationship is not causal
3	Non-analytical studies (e.g., case reports, case series)
4	Expert opinion, formal consensus

**Table 4:** NICE hierarchy of evidence and recommendation grading scheme

Level	Type of evidence	Grade	Evidence
1	Evidence obtained from a single randomised controlled trial or a meta-analysis of randomised controlled trials	A	At least one randomised controlled trial as part of a body of literature of overall good quality and consistency addressing the specific recommendation (evidence level I) without extrapolation
2	Evidence obtained from at least one well-designed controlled study without randomization or Evidence obtained from at least one other well-designed quasiexperimental study	B	Well-conducted clinical studies but no randomised clinical trials on the topic of recommendation (evidence levels II or III); or extrapolated from level I evidence
3	Evidence obtained from well-designed non-experimental descriptive studies, such as comparative studies, correlation studies		
4	Evidence obtained from expert committee reports or opinions and/or clinical experiences of respected authorities	C	Expert committee reports or opinions and/or clinical experiences of respected authorities (evidence level IV). This grading indicates that directly applicable clinical studies of good quality are absent or not readily available

**Fig. 1:**
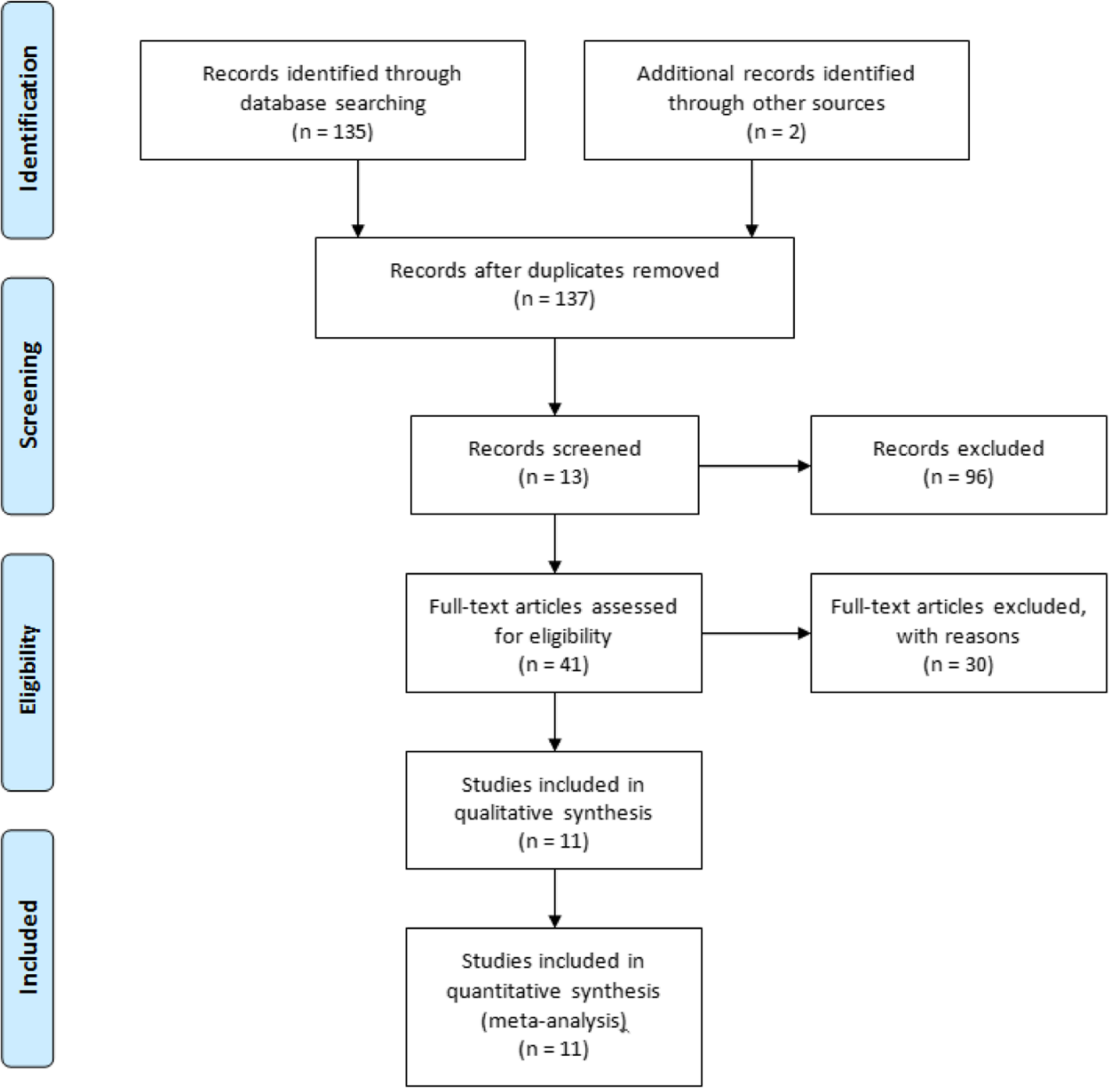
– Flow Diagram of screening process

The studies were further rated based on their scientific methodology and robustness using a Modified Coleman Methodology Scoring Tool on a scale of 0–100 [17] Fig. 1.

A quality assessment is vital within a systematic review in order to substantiate reliability and both internal/external validity of its findings and this tool is specifically designed to score a published study based on its methodological strengths [16]. All studies were automatically allocated the maximum score for follow-up due to the nature of the intervention/study outcomes, in line with NICE definitions of Surgical Site Infection [2]. This was deemed appropriate as with such antibiotic chemoprophylatic studies, there is no scientific and or clinical benefit for an extended long-term follow-up of more than 2 months, as an infection outside this date range would not be classed as an SSI [2].

## Results

Overall 11 studies met the inclusion criteria. From the grading of evidence, 2 level one and 4 level two studies were recognised. These studies ranked relatively highly in comparison to 5 studies that were graded as level three and level four tiers of evidence [2] Table 5.

**Table 5:** Classification of evidence based on NICE protocol [2]

Study	Study Design	Level of Evidence
Akinyoola et al. (2011) [19]	Prospective RCT	1-
Butterworth et al. (2017) [18]	Prospective Cohort Study	2++
Dayton et al. (2015) [34]	Consensus Panel (Expert Opinion)	4
Deacon et al. (1996) [20]	Prospective Cohort Study	2++
Dounis et al. (1995) [21]	Prospective Cohort Study	2-
Kurup (2016) [35]	Retrospective Cohort Study (Abstract Only)	4
Mangwani et al. (2016) [27]	Prospective RCT	1++
Pace et al. (2016) [36]	National Survey	4
Reyes et al. (1997) [37]	Retrospective Comparative Study	3
Tantigate et al. (2016) [28]	Retrospective Comparative Study	3
Zgonis et al. (2004) [22]	Retrospective Cohort Study	2+

Further data analysis summarised in Tables 6, 7. was undertaken using the PICO tool [15], evidenced a trend toward heterogeneity amongst the studies meeting the inclusion criteria, meaning it was not entirely possible to undertake pooled statistical analyses of the overall findings

**Table 6:** Modified Coleman Methodology Scoring Tool analysis of studies meeting inclusion criteria further demonstrated that over half of the studies included in the review scored 70 or over

Study	Study size	Mean f/u	Percentage of patients with f/u	No. interventions per group	Type of study	Diagnostic certainty	Description of surgical technique	Description of postoperative rehabilitation	Outcome criteria	Procedure for assessing outcomes	Description of subject selection process	Total
Akinyoola et al. (2011) [19]	106 (7)	5	100% (5)	1 (10)	Prospective (10)	All (5)	Technique named with detail (5)	Not reported (0)	2,2,3,3	5,0,3,3	0,5,0	73/100
Butterworth et al. (2017) [18]	4238 (10)	5	98% (5)	5	Prospective (10)	All (5)	Technique named (3)	Not reported (0)	2,2,3,3	0,0,3,3	5,5,5	74/100
Dayton et al. (2015) [34]	64 (4)	5	0	0	Retrospective (0)	0	0	0	2,2,0,0	5,0,0,0	5,0,0	23/100
Deacon et al. (1996) [20]	25 (4)	5	100% (5)	1 (10)	Prospective (10)	All (5)	Technique named with detail (5)	Not reported (0)	2,2,3,3	5,0,0,3	5,0,5	72/100
Dounis et al. (1995) [21]	67 (7)	5	100% (5)	2 interventions per group (5)	Prospective cohort (10)	Confirmed lab results (5)	Technique named (3)	(5)	2,2,3,3	5,4,4,0	5,5,5	83/100
Kurup (2016) [35]	340 (10)	5	100% (5)	1 (10)	Retrospective (0)	Confirmed lab results (5)	Technique named (3)	Not reported (0)	2,2,0,0	0,0,0,0	0,0,0	37/100
Mangwani et al. (2016) [27]	100 (7)	5	100% (5)	1 (10)	Prospective RCT (15)	All (5)	Technique named (3)	Not reported (0)	2,2,3,3	5,4,3,3	5,5,5	90/100
Pace et al. (2016) [36]	64 (4)	5	0	0	0	0	0	0	2,2,0,0	5,0,0,0	5,0,0	23/100
Reyes et al. (1997) [37]	459 (10)	5	100% (5)	Not stated (0)	Retrospective (0)	All (5)	Not stated (0)	Not reported (0)	2,2,0,0	0,0,0,3	5,5,0	42/100
Tantigate et al. (2016) [28]	1632 (10)	5	100% (5)	1 (10)	Retrospective (0)	Not stated (5)	Not stated (0)	Not reported (5)	2,2,3,3	5,0,0,3	5,5,5	73/100
Zgonis et al. (2004) [22]	555 (10)	5	100% (5)	1 (10)	Retrospective (0)	All (5)	Technique named (3)	Not reported (0)	2,2,3,3	5,0,0,3	5,5,5	71/100

The remaining studies were included based on their clinical relevance and to validate the paucity within the literature

**Table 7:** PICO Analysis for data extraction of the studies meeting the inclusion criteria [15]

Study Name	Akinyoola et al. (2011) Prospective RCT [19]
Participants	• 106 consecutive patients undergoing ‘clean, elective foot and ankle surgery’ although over 74% were trauma cases e.g. open fracture requiring open reduction and internal fixation (ORIF) thus diminishing the validity of measure as the researchers are no longer measuring what the study sets out or claims. • Surgery performed within five days of admission, meaning the patients were kept in hospital prior to and after surgery, which could raise the infection rates due to the potential to be colonised before and after surgery on the ward, around other unwell or infected patients. • Surgery performed using a thigh tourniquet not ankle.
Intervention	• Perioperative timing of antibiotic delivery. • IV Cefuroxime – did not state specific doses just ‘adjusted for age/weight’. • Researchers also gave patients up to three postoperative doses of IV antibiotics.
Control	• Group 1: Antibiotics received 5 min before inflation of tourniquet (ABT). • Group 2: Antibiotics received 1 min after inflation of tourniquet (AAT).
Outcomes	• Statistically significant postoperative infection rates of 14.8% (ABT) and 3.9% (AAT). • Operating surgeons (main researchers) determined the presence of infection thereby introducing experimenter bias at this stage in the study which may affect the internal validity of the findings.
Study Name	Butterworth et al. (2017) Prospective Cohort Study [18]
Participants	• 4238 patients who underwent foot and ankle surgery. • Prospectively rather than retrospectively reviewed. • Multicentre across Australia therefore high level of generalisability. • No randomisation, patients received or did not receive antibiotics dependant on their medical status, outlined procedure and surgeon’s decision. Therefore, patients who received postoperative antibiotics may have been of a less stable ASA Grading.
Intervention	• Patients receiving no antibiotics. • Patients receiving only preoperative antibiotics. • Patients receiving only postoperative antibiotics. • Patients receiving both pre-& postoperative antibiotics. • No mention of antibiotic type e.g. IV or Oral or agent. • No stratification of procedure location or type e.g. rearfoot or forefoot; fusion, osteotomy or toenail avulsion. • Cannot confirm whether patients actually took postoperative antibiotics.
Control	• No control owing to study design.
Outcomes	• Postoperative infection rates primary measure using an established and validated tool to capture data. • Comparisons amongst ASA Grade, age, gender, surgeon experience as predictors of infection. The latter being the only predictor with less rate of SSI with more experienced surgeons, perhaps likely due to a shorter duration of surgery with more experienced hands. • No antibiotics (2.5%). • Preoperative antibiotics (1.1%). • Postoperative antibiotics (2.6%). • Pre-& postoperative antibiotics (2.1%).
Study Name	Dayton et al. (2015) Consensus Panel (Expert Opinion) [34]
Participants	• 5 member panel of expert foot and ankle surgeons who received an invitation from the American College of Foot and ankle Surgery (ACFAS) to form part of a consensus group.
Intervention	• The panel worked to evaluate via email, telephone, face to face discussions the pertinent literature to antibiotic prophylaxis in foot and ankle surgery and found only 6 studies met their inclusion criteria after their literature search. • A modified Delphi method via a Likert Scale technique was then utilised to reach a consensus upon 13 pressing clinical questions put together by the panel chair.
Control	• No control owing to study design.
Outcomes	*Panel Consensus:*
	• It is appropriate to give antibiotic prophylaxis within foot and ankle surgery where the procedure involves bone, hardware and prosthetic joints. • It is generally accepted that patients at an increased risk of infection should be considered for antibiotic prophylaxis and that patient factors drive the decision to provide chemoprophylaxis over the type of procedure being performed, unless the type of procedure is considered to be a prolonged case (e.g. over 90 min). • Narrow spectrum antibiotics covering *Staphylococcus aureus* should be used for antibiotic prophylaxis in patients without a history of resistant infection. • It is not always appropriate to routinely perform preoperative nasal swabs to check for MRSA colonisation. • In cases where chemoprophylaxis is deemed necessary, antibiotics should be administered within 60 min prior to tourniquet inflation and discontinued within 24 h after surgery.
Study Name	Deacon et al. (1996) Prospective Cohort Study [20]
Participants	• 25 ASA 1 patients undergoing ‘bunionectomy procedures’ under the age of 55 with no previous history of ‘bunionectomy’ upon the same foot. • No explanation or stratification of type of procedure i.e. was metalwork used?
Intervention	• IV administration of 1 g of Cefazolin 60 min prior to tourniquet inflation. • Evaluated concentration of drug within 1st metatarsal head and compared to minimum inhibitory concentration required for inhibition of 90% of *Staphylococcus* taph Aureus in vitro (MIC90)
Control	• No control group utilised.
Outcomes	• IV administration of 1 g of Cefazolin led to mean bone concentration levels of 2.39 ± 1.19 (sd) μg/g. • This translates to approximately two to four times the required amount to exert a bacteriostatic effect at the site of surgery (0.5–1.0 μg/g required MIC90). • This was not a study to assess postoperative infection rates, however no infections were encountered. • Researchers concluded that 1 g of IV Cefazolin 60 min prior to tourniquet inflation will provide bone concentration levels of antibiotic that are adequate in principle to inhibit colonization *Staphylococcus* taph Aureus*.* • This study used ASA 1 patients therefore findings are based on the assumption that an unremarkable medical history and peripheral vascular status, with adequate circulation will achieve the required concentration levels as serum concentration was not individually measured in this study.
Study Name	Dounis et al. (1995) Prospective Cohort Study [21]
Participants	• 67 patients undergoing foot and ankle surgery with no hepatic/renal insufficiency, known allergies to cephalosporin/penicillin antibiotics or concurrent steroid/immunosuppressant/antibiotic therapy. • Procedures included: 10 ankle fusions, 7 triple arthrodesis of rearfoot, 12 Keller’s arthroplasties, 15 Chevron osteotomies, 16 Mitchell osteotomies and 7 metatarsal head excisions on patients ranging from 39 to 72 with a mean age of 58.9 years old ±9.2 (sd). • All procedures carried out using thigh tourniquet.
Intervention	• Systemic IV vs Local IV Antibiotics (distal to tourniquet). • Intervals of 10 min, 20 min, 2 h and 4 h prior to tourniquet inflation in the systemic group. • Ceftazidime 2 g vs Ceftriaxone 2 g in each group although in the local group were mixed with 25 ml of 0.9% Saline to “fill empty intravascular space” no details of how they calculated weight/height to reach this figure, negatively affects internal validity making it difficult to draw comparisons as antibiotics preparations not controlled for. • Evaluated bone and soft tissue concentrations of participants 10–15 min after skin incision.
Control	• Group 1: Antibiotics received IV systemic at stratified intervals of 10 min, 20 min, 2 and 4 h prior to tourniquet inflation. • Group 2: Antibiotics received IV local after tourniquet inflation but no record of time was described or which distal vein was utilised within methodology.
Outcomes	• Ceftazidime 2 g & Ceftriaxone 2 g demonstrated similar bone and soft tissue concentrations in each group. • In the systemic IV group the highest concentration of antibiotic in bone & soft tissue was yielded 20 min before tourniquet application. • In the local IV group the concentration of antibiotic in bone and soft tissue was consistently between 4 and 12 times higher than systemic IV administration (*P* > 0.001).
Study Name	Kurup (2016) Conference Paper Abstract Only [35]
Participants	• 340 patients undergoing elective forefoot surgery where perioperative antibiotics were deemed necessary by the operating clinician were reviewed retrospectively between 2011 and 2015. • Procedures ranged from digital fusion, osteotomy and joint replacement. • No description of ASA grade or antibiotic regimen/timings.
Intervention	• Procedures “involving metal/resorbable implant or prosthesis” were undertaken with patients receiving 400 mg of IV Teicoplanin.
Control	No control owing to study design.
Outcomes	• No cases of deep infection. • 1.7% infection rate with 6 superficial soft tissue infections recorded. • Due to nature of publication i.e. abstract only- high level of detail lacking.
Study Name	Mangwani et al. (2016) Prospective RCT [27]
Participants	• 100 patients undergoing lesser toe fusion in which an external k-wire was to be left in situ post-surgery for 4–6 weeks, with a mean age of 58 (group one) ± 17.5 (sd) and 62.7 (group two) ± 14.7 (sd) years old. • No stratification for ASA grade/co-morbidities.
Intervention	• Stratified random allocation of prophylactic Flucloxacillin (or Teicoplanin where an allergy was present). • No description or detail of timing/dose/route other than administration remained consistent with operating clinician’s standard preferences.
Control	• Group 1: Received prophylactic antibiotics • Group 2: Did not receive prophylactic antibiotics
Outcomes	• Group 1: 3 infections (6.2%) 93 toes, 4 diabetics + 1 immunosuppressed patient on methotrexate for sero-negative spondyloarthropathy (PSA) with a high BMI. • Group 2: 1 infection (1.9%) 78 toes, 2 diabetics. • Results not found to be significant after statistical analysis. • Difference in % of patients more susceptible to infection across 2 groups may account for study findings and may not accurately predict likelihood of infection rate with/without antibiotic prophylaxis for this type of surgery, acknowledged by the researchers.
Study Name	Pace et al. (2016) National Survey [36]
Participants	• 112 Orthopedic clinicians from the US were mailed a survey with respect to foot surgery and the use of antibiotics and percutaneous k-wires. • A total of 64 (57%) completed the survey with a mean of 15.2 years post fellowship acquisition.
Intervention	• Mail survey consisted of three clinical scenarios pertaining to use of percutaneous k-wire fixation and minimising postoperative pin tract infection: non-diabetic, diabetic, diabetic with sensory neuropathy. • Evaluated duration of k-wire placement, whether postoperative antibiotics were considered appropriate and which antibiotics are routinely used. • No questions evaluating the use of preoperative antibiotics.
Control	No control owing to study design.
Outcomes	• First case: 25% would use postoperative antibiotics, mean 9.4 days. • Second case: 28% would use postoperative antibiotics, mean 13.8 days. • Third case: 32% would use postoperative antibiotics, mean 14.5 days. • Majority of clinicians would utilise oral Cephalexin postoperatively. • None of the findings were shown to be statistically significant in this study.
Study Name	Reyes et al. (1997) Retrospective Review [37]
Participants	• 459 cases of foot and ankle surgery were reviewed between 1993 and 1996. • Data was gathered on the use of antibiotics, tourniquet, anaesthetic and patient demographics/medical history and type of surgery in order to explore postoperative infection rate and trends in practice.
Intervention	• The following data was recorded from patient records and used for descriptive analysis: antibiotic prophylaxis, age, gender, medical history, procedure duration, fixation used, tourniquet use and postoperative infection. • Majority of cases used Cefazolin 1 g or Vancomycin in those allergic.
Control	No control owing to study design.
Outcome	• Overall infection rate of 0.65%. • Infection rate with antibiotic prophylaxis was 0.43%. • Infection rate without antibiotic prophylaxis was 0.88%. • Not found to be statistically significant. • 50.7% of cases received IV antibiotics 30–60 min prior to surgery, the remaining did not receive any antibiotics. • 82% of cases involving bone and internal fixation were dosed with preoperative antibiotics. • Average tourniquet time of 68.72 min used at ankle and thigh. • No description of type of surgery/co-morbidities/ASA grade etc.
Study Name	Tantigate et al. (2016) Retrospective Comparative Study [28]
Participants	• Retrospective chart review of 1933 ft and ankle procedures in 1632 patients over a 56 month period.
Intervention	• Demographic data, type of antibiotics/dosage/timing were recorded along with rate of postoperative infection.
Control	No control owing to study design.
Outcomes	• When antibiotics were administered between 15 and 60 min prior to incision, there was a 2.7 – fold, statistically significant higher rate of postoperative infection as compared to the group of patients who received antibiotics< 15 min before incision (*P* < 0.05). • Independent predictors of postoperative infection were ASA Grade, non-ambulatory surgery and lengthier duration of surgery, with almost 92% of the risk of a postoperative infection being predicted by these factors (*P* > 0.05). • Suggests host factor may play a bigger role in predicting risk of postoperative infection than timing of antibiotics, though did not control for these host factors in a prospective and/or randomised manner therefore difficult to draw any causal relationship, moreover no description of type of antibiotics/timing/dose/regimen/type of surgery or ASA grade in abstract published.
Study Name	Zgonis et al. (2004) Retrospective Review [22]
Participants	• 555 patients who received elective foot and ankle surgery between 1995 and 2001. • Patients who had prior ulcerations, infection or trauma to the foot and ankle were excluded. • Patients were stratified into 6 categories: soft-tissue, digital, lesser metatarsal, first ray, rearfoot and multiple surgeries (more than one procedure).
Intervention	• The following data was recorded from patient records and used for statistical analysis: antibiotic prophylaxis, age, gender, medical history, procedure duration, fixation used, tourniquet use and postoperative infection.
Control	No control owing to study design.
Outcome	• IV antibiotics for prophylaxis were stated to be ordered 30 min prior to incision but administration varied from between 2 h prior to incision and just before surgery, meaning that either there has been administration before the drug order was put in or an error in the recording. Nonetheless, these are the details documented in the researcher’s findings, meaning that unfortunately this was not standardised or consistent, which is unfortunately a common attribute of the study design utilised. • The overall infection rate was 3.1%. • 55.1% of patients received antibiotic prophylaxis in this study with an infection rate of 1.6%. • 44.9% of patients did not receive antibiotic prophylaxis and rate of infection was found to be 1.4% postoperatively. • This was not deemed to be statistically significant, though to demonstrate this the power of the study would need to be significantly improved with a considerably larger sample size of 3452 patients for each group (receiving/not receiving antibiotics) totalling almost 7000 patients. This would be challenging to undertake both practically and ethically with certain surgical institutions having to follow local hospital policy and therefore recruiting over 3000 patients into one of the study groups which would then receive an intervention which goes against local policy may prove an arduous task to justify.

### Infection incidence

Results of SSI rates found within this systematic review ranged from 0 to 9.4% of overall postoperative infections encountered after foot and ankle surgery in the studies analysed Table 8.

**Table 8:** Comparison of SSI rates within all studies utilised for this systematic review

Study	# Participants	Incidence of SSI	
		*Antibiotic Prophylaxis*	*No Antibiotic Prophylaxis*
Akinyoola et al. (2011) [19]	106	9.4%	N/A
Butterworth et al. (2017) [18]	4238	1.1%	2.6%
Dayton et al. (2015) [34]	N/A	N/A	N/A
Deacon et al. (1996) [20]	25	0%	N/A
Dounis et al. (1995) [21]	67	N/A	N/A
Kurup (2016) [35]	340	1.7%	N/A
Mangwani et al. (2016) [27]	100	6.2%	1.9%
Pace et al. (2016) [36]	N/A	N/A	N/A
Reyes et al. (1997) [37]	459	0.43%	0.88%
Tantigate et al. (2016) [28]	1632	1.1%	N/A
Zgonis et al. (2004) [22]	555	1.6%	1.4%

A multicentre prospective cohort study of 4238 patients [18] demonstrated that without antibiotic prophylaxis infection rate was 2.6% which was 1.5% higher than the group receiving chemoprophylaxis which was 1.2% incidence. Interestingly, they also reviewed a separate cohort which received both pre-& postoperative antibiotics who demonstrated an SSI rate of 2.1%.

After collating the information upon those studies which compared chemoprophylaxis vs no chemoprophylaxis the total pooled data demonstrated postoperative infection rates of 1.2% (antibiotic chemoprophylaxis) and 2.3% (no antibiotic chemoprophylaxis) (Table 9).

**Table 9:** Overview of studies which compared SSI rates in cohorts which received and did not receive antibiotic prophylaxis

Study	# Participants	Incidence of SSI		Overview	
		*Antibiotic Prophylaxis*	*No Antibiotic Prophylaxis*	Study Design	Level of Evidence
Butterworth et al. (2017) [18]	4238	1.1%	2.6%	Prospective Cohort Study	2++
Mangwani et al. (2016) [27]	100	6.2%	1.9%	Prospective RCT	1++
Reyes et al. (1997) [37]	459	0.43%	0.88%	Retrospective Cohort Study	3
Zgonis et al. (2004) [22]	555	1.6%	1.4%	Retrospective Cohort Study	2+

### Incidence of adverse drug event

From analysis of all studies, there was no reported incidence of anaphylaxis, toxicity or any other adverse drug event and/or serious side effects related to the use of antibiotics in foot and ankle surgery Table 10.

**Table 10:** Pooled data from the 4 studies which drew causal relationships between antibiotic chemoprophylaxis and subsequent SSI rate

# Participants	Incidence of SSI	
	*Antibiotic Prophylaxis*	*No Antibiotic Prophylaxis*
5352	64 (1.2%)	124 (2.3%)

### Pneumatic tourniquet application and antibiotics

Three studies evaluated antibiotic chemoprophylaxis and the influence of tourniquet application [19–21]. One RCT prospectively evaluated SSI rates and the delivery of antibiotics both 5 min prior to tourniquet inflation and 1 min after inflation [19]. This study reported infection rates of 14.8% (antibiotics before inflation) and 3.9% (antibiotics after inflation) respectively within the groups studied.

Two studies prospectively evaluated antibiotic bone penetration, using the minimum inhibitory concentrations (MIC90) as a quantitative marker to demonstrate effectiveness against 90% of the most common causative organisms found in vitro at the site of surgery [20, 21].

A prospective cohort study of 25 patients reported that IV administration of 1 g Cefazolin 60 min prior to tourniquet inflation led to mean bone concentration levels of 2.39 ± 1.19 μg/g (sd).

This translates to approximately 2 to 4 times the required amount to exert a bacteriostatic effect at the site of surgery (0.5–1.0 μg/g required MIC90) [20] raising the question of whether this standard dose of IV Cephalosporin is unnecessarily high and in the course of good antimicrobial stewardship whether there would be any benefit to reducing this dose where chemoprophylaxis is required Tables 11 and 12.

**Table 11:** Use of Antibiotics in studies meeting the inclusion criteria and their administration protocol where defined

Study	Antibiotic Agent(s)	Dose	Route	Timing
Akinyoola et al. (2011) [19]	Cefuroxime	Not stated	IV	5 min prior or 1 min following tourniquet inflation as well as 3 doses postoperative at 8 h intervals
Butterworth et al. (2017) [18]	Not stated	Not stated	Not stated	Not stated
Dayton et al. (2015) [34]	N/A	N/A	N/A	N/A
Deacon et al. (1996) [20]	Cefazolin	1 g	IV	34–60 min prior to incision
Dounis et al. (1995) [21]	Ceftazidimine or Ceftriaxone	2 g	IV	10–240 min prior to inflation of tourniquet
Kurup (2016) [35]	Teicoplanin	400 mg	IV	Not stated
Mangwani et al. (2016) [27]	Flucloxacillin or Teicoplanin	Not stated	Not stated	Not stated
Pace et al. (2016) [36]	N/A	N/A	N/A	N/A
Reyes et al. (1997) [37]	Cefazolin or Vancomycin	Not stated	IV	30–60 min according to manufactures guidance prior to incision
Tantigate et al. (2016) [28]	Cefazolin, Clindamycin or Vancomycin	Not stated	IV	0–60 min prior to incision
Zgonis et al. (2004) [22]	Not stated	Not stated	IV	0–120 min prior to incision

**Table 12:** List of pathogens encountered in those patients who developed a postoperative SSI in the study conducted by Zgonis et al. [22]

Pathogen	Sensitivity	Frequency
Coagulase-negative *Staphylococcus epidermidis*	Penicillin Resistant Ampicillin Resistant	6
Coagulase-positive *Staphylococcus Aureus*	Penicillin Resistant Ampicillin Resistant	6
Coagulase-positive *Staphylococcus Aureus*	Penicillin Sensitive Ampicillin Sensitive	2
Methicillin-resistant *Staphylococcus Aureus*	Penicillin Resistant	1
*Peptostreptococcus*	Penicillin Resistant	1
Beta haemolytic *Streptococcus*	Penicillin Resistant	1
*Enterobacter cloacae*	Penicillin Resistant	1
*Pseudomonas Auriginosa*	Penicillin Resistant	1
Gram-positive *cocci*	Penicillin Resistant	1
*Alcaligenes faecalis*	Penicillin Sensitive	1

### Causative organisms

Zgonis et al. [22] examined bacterial isolates of patients with laboratory confirmed SSI following elective foot and ankle surgery, concluding that the most commonly encountered pathogens were coagulase negative and positive *Staphylococcus.* Interestingly, all patients with preoperative chemoprophylaxis and postoperative wound infection developed a pathogen unfailingly resistant to Penicillin and/or Ampicillin. These findings are consistent with similar studies which have noted a proliferation in difficult to treat bacterial isolates of Methicillin-esistant *Staphylococcus aureus and epidermidis* (MRSA/MRSE) and Vancomycin resistant *Staphylococcus aureus and Enterococcus* (VRSA/VRSE) in clean elective surgery [23] [24–26]. Thus validating the thought that unremitting overuse of the same prophylactic antibiotic(s) will result in the evolution of tremendously virulent pathogenic isolates, which will arduously be a challenge to treat postoperatively [18, 22, 27].

### Duration and type of foot and ankle surgery

Zgonis et al. demonstrated a statistically significant association (*P* < 0.001) between antibiotic use and surgical category and fixation choice [22]. However, as patients were not randomised prospectively owing to the nature of the study, patients undergoing more complex surgery with higher amounts of both internal and external fixation were more likely to receive antibiotics due to surgeon preference.

This could therefore explain the difference in infection rates between the 2 groups (antibiotic vs no antibiotic) as a longer duration of surgery with higher amount of implanted fixation may indeed lead to a higher rate of infection and it would be these patients who received preoperative antibiotics, had they not been prophylaxed it could be argued that the infection rate could have been higher in this group.

### Risk & predictive factors for SSI

No studies appraised type and duration of foot and ankle surgery as independent variables influencing SSI rates, with the exception of Tantigate el al. postulating via the findings in their study, that independent predictors of postoperative infection were ASA Grade, non-ambulatory surgery and a longer duration of surgery, with almost 92% of the risk of a postoperative infection being predicted by these factors (*P* > 0.05) alone [28].

### Summary of studies

The characteristics of the research represented seems to be split into a mixture of largely retrospective or prospective cohort designs, with more recent studies adopting a prospective approach and recognising the need for better quality research into the area. All studies reviewed were either published in the 1990s or post 2010, indicating a lag-period of nearly 20 years where the topic was not well researched. These studies were international and no one country seems to be leading upon this research field.

## Discussion

### Infection incidence

The results of the systematic review demonstrate inconsistent postoperative infection rates, ranging from 0 to 9.4% of overall SSI’s encountered after foot and ankle surgery.

The highest-level study enrolled 100 patients [27]. With this type of prospective RCT a sample size between 425 and 1145 could be used to determine a reduction of infection from 4 to 1% and 4% to 2% assuming 80% power and statistical significance of 5%, although it may be possible that even with a larger sample size the results may not differ significantly. However, this study was unique in that it exclusively reviewed lesser toe surgery and not other types of foot and ankle surgery; moreover, with the use of percutaneous k-wires in all cases, the application or generalisability of these findings to studies which do not utilise external fixation methods may be limited.

A multicentre prospective cohort study of 4238 patients [18] demonstrated significantly higher infection rates when patients received both pre and postoperative antibiotics in comparison to those who received preoperative antibiotics alone, concluding that postoperative antibiotics are counter-productive for clean elective foot and ankle surgery.

### Influence of pneumatic tourniquet application

Pragmatically, in the manifestation of transitory surgical limb ischaemia, the MIC90 of an antibiotic may diminish at the operative site, a hypothesis which would suggest that prophylaxis would then be less effectual. There is however, deficient evidence to sustain this rationale within the extant latitude of literature.

A prospective RCT of 106 consecutive patients undergoing ‘clean, elective, foot and ankle surgery’ demonstrated SSI rates of 14.8% (Group 1) and 3.9% (Group 2) in 2 patient cohorts, both of whom received IV antibiotics. Group 1 received IV Cefuroxime 5 min prior to tourniquet inflation and Group 2 were administered chemoprophylaxis 1 min after inflation in an attempt to discern thigh tourniquet influence upon postoperative infection rate [19]. The researchers, however, admitted that over 74% of procedures were trauma cases e.g. open fractures requiring ORIF and up to a 5 day inpatient stay as standard practice at this medical facility. This would suggest that the internal validity of this RCT is compromised as it is no longer evaluating infection rates within ‘clean, elective, foot and ankle surgery’ as the study had outlined in its design and aims.

### Antibiotic regimen

All studies within this systematic review which described their antibiotic regimen utilised exclusively IV antibiotics, with the majority of studies utilising Cephalosporin antibiotics [19–21, 28].

However, there has been a national drive within the UK particularly, to move away from this type of antibiotic for the use of surgical prophylaxis not only to mitigate the critical risk of bacterial resistance but also to subdue the rates of *Clostridium difficile (C.diff)* infections in the hospital environment [29, 30]. According to the NICE guidelines (2015) the results of 3 meta-analyses established that the most prominent antibiotics associated with nosocomial *C.diff* infection were second and third generation Cephalosporin’s, Clindamycin and the Fluoroquinolone group [30]. This makes the development of local guidelines challenging due to the existing body of evidence being largely based on the use of a class of antibiotics which are believed to be broad not narrow spectrum drugs.

Moreover, the Cephalosporin group has been shown to be associated with higher rates of *C.diff* infection and are consequently being discouraged as a primary agent for surgical prophylaxis in clean elective surgery by some health authorities, therefore going against the national UK guidelines it seems on not one - but two - fronts [4, 29, 30]. It would seem pertinent in light of this issue to undertake further prospective studies evaluating the use of narrow spectrum antibiotics which are not associated with the reported higher rates of *C.diff* infection.

### Oral vs. IV

The most prominent authority guidelines on the use of antibacterial chemoprophylaxis counsel on the use of the IV [2–4]. Oral antibiotic drugs must first be metabolised via the bodies first pass mechanism, increasing the length of time taken to reach MIC90, which should be reached by the first incision in order for chemoprophylaxis to be most effective [3, 20]. In the case of oral administration of antimicrobials, there is some high level evidence to suggest oral antibiotic prophylaxis is as effective as IV prophylaxis [31], though no studies specific to the foot and ankle have been published to date. It is suggested that antibiotic chemoprophylaxis take place within 60 min prior to tourniquet inflation [32, 33]. The significant lack of relevant research into oral antibiotic prophylaxis is noted and once again, prospective study of the RCT design is warranted in order to validate the widely held hypothesis that IV administration of antibiotics for surgical prophylaxis is more effective than oral or intramuscular (IM) routes [2–4].

### Bolus vs. multiple doses

Evidence yielded from this systematic review further proposes that a solitary dose of prophylactic antibiotics, with a suitable half-life, is appropriate in diminishing postoperative infection rate [2]. Re-dosing is recommended in surgical procedures lasting more than 2 half-lives of the antibiotic used for surgical prophylaxis with consideration to blood loss (> 1.5 L), fluid replacement and patient factors e.g. both liver and renal function [2, 3]. It is noted however that from conducting this review no studies in foot and ankle surgery have independently reviewed the influence of re-dosing at various time intervals intra-operatively and ensuing outcomes related to SSI.

### Narrow Spectrum antibiosis

It should be noted that *Staphylococcus aureus and Staphylococcus epidermidis* are common sources of infection in foot and ankle surgery and are particularly associated with infections of foreign material due to their propensity to form biofilms [2–4, 22].

The choice of suitable antibiosis is of paramount importance to safeguard absolute efficiency while curtailing the risk of adverse drug events, which was interestingly not reported in any of the studies in this systematic review. If antibacterial prophylaxis is used, the agent(s) chosen should be narrow spectrum and avoid any unnecessary broad-spectrum activity [2, 4].

## Conclusion

### Summary of recommendations

- In clean, uncomplicated and elective soft tissue surgery of the foot and ankle antibiotic prophylaxis is not routinely warranted [2–4, 18–22, 27, 28, 34–37].
*Grade of recommendation: B.*
- In clean, uncomplicated and elective bone and joint surgery of the foot and ankle antibiotic prophylaxis is not routinely warranted [2–4, 18–22, 27, 28, 34–37].
*Grade of recommendation: B.*
- Where the use of metallic hardware is necessitated, the decision to provide prophylaxis must be agreed locally as a conclusive recommendation on whether or not to chemoprophylax cannot be made due to the paucity of the literature in favour of chemoprophylaxis within foot and ankle surgery. With respect to this, the decision should ultimately be based upon surgeon preference, patient and surgical risk factors with pertinence to the amount and type of hardware and surgery required. It is the personal view of the author that until better research is established national guidelines be followed and chemoprophylaxis in this situation be provided [2–4, 18–22, 27, 28, 34–37]
*Grade of recommendation: C.*
- Where the use of joint replacement prostheses are necessitated, antibiotic prophylaxis is recommended due to their propensity to form biofilms [2–4, 18–22, 27, 28, 34–37].
*Grade of recommendation: A.*
- Antibiotic prophylaxis should be delivered to the patient within 60 min of inflation of pneumatic tourniquet to allow sufficient plasma concentrations of the drug to be utilised [2–4, 18–22, 27, 28, 34–37].
*Grade of recommendation: A.*
- There is insufficient current evidence to suggest that IV antibiotics are more effective than oral or IM with respect to chemoprophylaxis in foot and ankle surgery, it is therefore advised that local policy considers drug class, local resistance patterns, drug penetration and bioavailability as well as being administered within a sufficient time frame to allow for adequate serum tissue concentrations to be reached (usually within 60 min) [18–22, 27, 28, 34–37].
*Grade of recommendation: C.*

### Strengths & Limitations

The strengths of this systematic review are the clear definition of the research question, which eliminates bias in the selection of the studies. Furthermore, the strengths of our study are the stringent adherence to an unambiguous research protocol that was developed prior to the analysis. As well as this, the broad nature of the literature search and consensus between the two reviewers throughout the screening process add validity and reliability to the research findings.

Nevertheless, despite the strengths of the review process, the authors concede that the absence of high level studies within the literature is apparent. The primary limitation of this review is that a direct comparative meta-analysis of infection rates within foot and ankle surgery, with and without antibiotic prophylaxis was not possible because there are few head-to-head trials.

Additionally, the variability of the outcomes measures, limited the number of studies which could be directly compared. Indeed, dissimilarities in patient cohorts, surgical procedures and variances in outcome assessment tools will be in some measure responsible for heterogeneity among these studies and prospective researchers should aim to address these issues with high level prospective studies.

### Recommendations for further research

There is a need for more prospective randomised studies within the field of foot and ankle surgery where drug route (IV, IM and oral) is utilised as an independent variable, currently no such study exists to inform the decision as to which route would be superior for foot and ankle surgery and we are therefore reliant on studies specific to more proximal (to the ankle) surgery.

Moreover, prospective randomised studies must also evaluate the influence of antibiotic chemoprophylaxis upon SSI rate in clean elective foot and ankle surgery where metallic hardware is implanted as internal fixation, as the available studies, national guidelines and UK national audit data all appear to represent a conflicting representation of views and warrant review.

## Abbreviations

ACFAS: American College of Foot & Ankle Surgeons 55
ASA: American Society of Anaesthesiologists
ASHP: The American Society of Health-System Pharmacists
C.Diff: Clostridium Dificile
IDSA: The Infectious Diseases Society of America
IM: Intramuscular
IV: Intravenous
NICE: National Institute for Health and Clinical Excellence
ORIF: Open Reduction Internal Fixation 56
PICO: Patient, Intervention, Control, Objective
SHEA: Society for Healthcare Epidemiology of America
SIGN: Scottish Intercollegiate Guidance Network
SIS: The Surgical Infection Society
SSI: Surgical Site Infection
